# Multi-criteria decision analysis framework for engaging stakeholders in river pollution risk management

**DOI:** 10.1038/s41598-024-57739-y

**Published:** 2024-03-26

**Authors:** Zesizwe Ngubane, Viktor Bergion, Bloodless Dzwairo, Thor Axel Stenström, Ekaterina Sokolova

**Affiliations:** 1 Department of Civil Engineering, Durban University of Technology, Pietermaritzburg, 3201 South Africa; 2 Department of Architecture and Civil Engineering, Chalmers University of Technology, 41296 Gothenburg, Sweden; 3 Institute for Water and Wastewater Technology, Durban University of Technology, Durban, 4000 South Africa; 4 Department of Earth Sciences, Uppsala University, 75105 Uppsala, Sweden

**Keywords:** Environmental sciences, Hydrology

## Abstract

Water pollution presents a substantial environmental challenge with extensive implications for water resources, ecosystem sustainability, and human health. Using a South African catchment, this study aimed to provide watershed managers with a framework for selecting best management practices (BMPs) to reduce pollution and the related risk to river users, while also including the perspectives of key catchment stakeholders. The framework encompassed the identification of and consultation with key stakeholders within the catchment. A Multi-Criteria Decision Analysis (MCDA) methodology using the Simple Multi-Attribute Rating Technique for Enhanced Stakeholder Take-up (SMARTEST) was used to identify and prioritise suitable BMPs in a case study. Decision alternatives and assessment criteria as well as their weights were derived based on stakeholder responses to a two-stage survey. Stakeholders included those utilising the river for domestic and recreational purposes, municipal representatives, scientists, NGOs, and engineers. The assessment of decision alternatives considered environmental, economic, and social criteria. The aggregated scores for decision alternatives highlighted the significance of involving stakeholders throughout the decision process. This study recommends the pairing of structural and non-structural BMPs. The findings provide valuable insights for catchment managers, policymakers, and environmental stakeholders seeking inclusive and effective pollution mitigation strategies in a catchment.

## Introduction

The issue of river pollution has become a concern in today's world, with detrimental effects on ecosystems^1^, human health^2^, and overall environmental well-being. The pollution of rivers may pose a significant threat to aquatic life, water quality, and the sustainability of natural resources. Addressing this requires urgent attention and effective measures to mitigate pollution sources, restore affected ecosystems, and ensure the long-term health and viability of rivers. The concept of catchment management strategies has been widely used to alleviate pollution^3^. These strategies, known as best management practices (BMPs), may include specific schedules, bans, guidelines, and other measures to prevent or reduce water pollution^4^. These BMPs have included structural interventions, for instance, constructed wetlands^1^, buffer strips, retention ponds^5^, or porous pavements^6^. They have also included non-structural BMPs, for instance, animal waste management^7^, advanced tillage systems^8^, nutrient management plans^8,9^, pesticide management plans^10^, planned grazing systems on pasture and rangeland^9^, erosion control^11^, and public education^9^. While the structural and non-structural management plans have been well studied, their implementation is often inhibited by limited resources, multiple conflicting criteria, cost effectiveness, and technical feasibility under specific circumstances^12,13^.

In order to successfully decrease river pollution, it is crucial to engage a diverse group of stakeholders who have an interest in or are impacted by changes in the catchment area. These stakeholders should come from various sectors, including social, policy, institutional, and financial domains. The collective aim of the stakeholders is to identify the most effective strategies for reducing pollution^4^. Effectively utilising the capabilities and dedication of stakeholders can greatly enhance the capacity to safeguard, develop, conserve, and manage water resources^14^. These stakeholders could be community-based organisations, water user associations, catchment management forums, non-governmental organisations (NGOs), academic and scientific communities, and the private sector^14^. Adom and Simatele^15^ conducted a study that confirmed the importance of stakeholder engagement in water resource management in South Africa for improving the collective comprehension of the decision and policy making process. This understanding plays a pivotal role in decision-making and has a positive influence on the sustainable management of water resources.

Multi-criteria decision analysis (MCDA) has found extensive application in water resource management. It offers a systematic and transparent approach that can be trusted to acknowledge stakeholder values^13^. For instance, MCDA has been utilised to prioritise vulnerable areas within catchments, evaluate ecosystem services^13^, facilitate stakeholder engagement^16^, and optimise rainwater harvesting strategies^17^. MCDA provides a comprehensive means to evaluate water resource management from a social, economic, technical, and ecological perspectives^18^. Given the wide range of available MCDA techniques, it becomes crucial to carefully evaluate the nature of the decision problem, the available data, the decision-maker's preferences, and the unique characteristics of each technique while selecting the most appropriate MCDA approach. Nevertheless, to maintain control and predictability, decision-making procedures in water resource projects have traditionally limited participation of civil society and favoured a small number of specialists^19^. This poses a challenge that needs to be addressed to ensure more inclusive and effective water resource management.

In the uMsunduzi catchment in South Africa (comprised of rural, urban, and informal settlements), a variety of water pollutants have been identified, including potentially toxic chemicals^20^ and pathogens^21^. Furthermore, the risk to human health has been quantified for pathogens^22^ and toxic chemicals^23^. In these studies, scenarios of ingestion through domestic and recreational uses (swimming and canoeing) were investigated, and the water was found unsuitable for these uses. This highlights the importance of alleviating pollution in the uMsunduzi River to reduce the effects on human health and the environment.

Using the uMsunduzi catchment in South Africa, this study aims to provide watershed managers with a framework for selecting BMPs to reduce pollution and the related risk to river users, while also including the perspectives of key catchment stakeholders. The specific objectives were to: (1) identify key stakeholders within the catchment; (2) consult the identified stakeholders to solicit BMPs to alleviate pollution and the BMP evaluation criteria; (3) apply the MCDA methodology to compare and prioritise the BMPs.

## Methods

### Case study area

**Figure 1:**
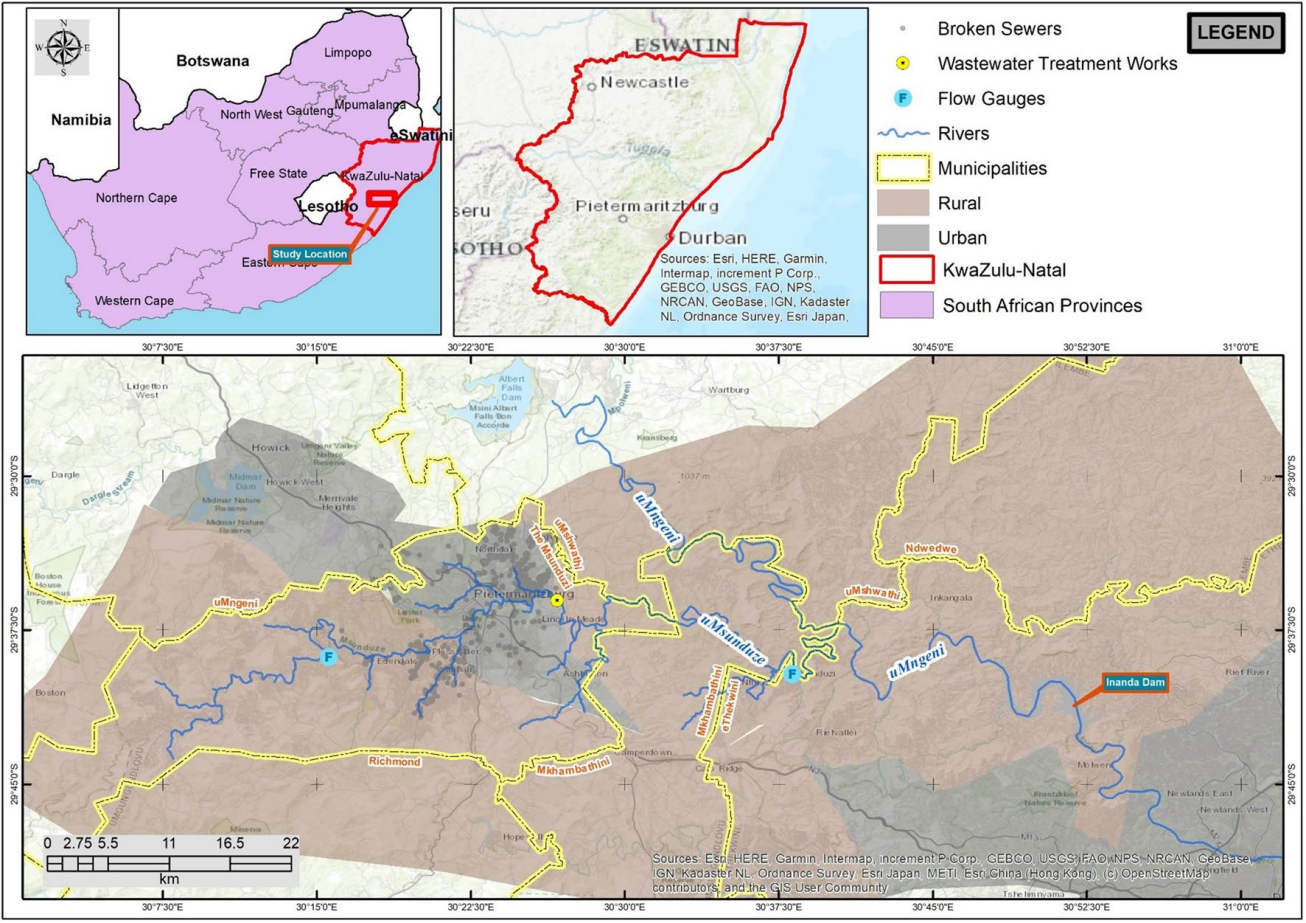
UMsunduzi catchment location. Please follow this link for an online Google Map.

UMsunduzi catchment is located in the province of KwaZulu-Natal in South Africa. The uMsunduzi River is a major tributary of the uMngeni River and contributes to Inanda Dam, which is one of the drinking water sources for the Durban Metropolitan area. The uMsunduzi River flows through rural and urban dwellings (Fig. 1) of Msunduzi Municipality (~54 600 population in 2023) and Mkhambathini Municipality (~63 200 population in 2023)^24^. Rural communities and informal settlement communities mainly use the water for domestic purposes and recreational swimming in warm months. Additionally, the uMsunduzi River is used for canoeing training and the annual Dusi Canoe Marathon in urban sections.

Risk of contracting illnesses during domestic and recreational activities was assessed through quantitative microbial and chemical risk assessment enacted by Ngubane et al.^22,23^. In Ngubane et al.^22^, the Soil and Water Assessment Tool (SWAT)^25^ and the quantitative microbial risk assessment (QMRA)^26^ were used to highlight areas of high microbial pollution and the risk that the water may pose to the health of consumers who use the river for domestic and recreational purposes. It was concluded that the uMsunduzi River is highly polluted with pathogens, and the use of untreated water from the river may result in a high risk of infection to exposed population^22^. The main faecal sources in the uMsunduzi catchment were summarised as: Darvill wastewater treatment plant, broken sewers in the urban area, and faecal droppings from grazing livestock. Investing in water treatment facilities, regulation of livestock practices, and safe sanitation systems for communities in need were suggested as solutions likely to provide sustainable and reliable improvement in the uMsunduzi River water quality.

In Ngubane et al.^23^, a quantitative chemical risk assessment (QCRA) was performed for organochlorinated pesticides (OCPs), pharmaceuticals and personal care products (PPCPs), heavy metals, and nitrates and phosphates in the context of the uMsunduzi River. It was concluded that the presence of OCPs posed risks of both carcinogenic and non-carcinogenic health effects in all subbasins and exposure scenarios. While other chemicals showed lower health risks, risks to the environment remain possible, and increases in chemical inputs would likely increase risk of illness to exposed population. The presence of excessive nutrients can cause harmful algae blooms by accelerating certain algae’s growth-decay cycle over others and therefore severely disrupt normal functioning of the ecosystem^12^, as has been the case for Inanda Dam in South Africa^27^. Activities such as subsistence farming, small plantations, illegal waste dumping, industries, broken sewers are some of the activities that potentially contribute to chemical pollution in the uMsunduzi River. Some suggested alleviation solutions were chemical control technologies for stormwater and household wastewater, as well as educating the public and businesses about the importance of protecting surface water from improper use, storage and disposal of pollutants^12^.

**Figure 2:**
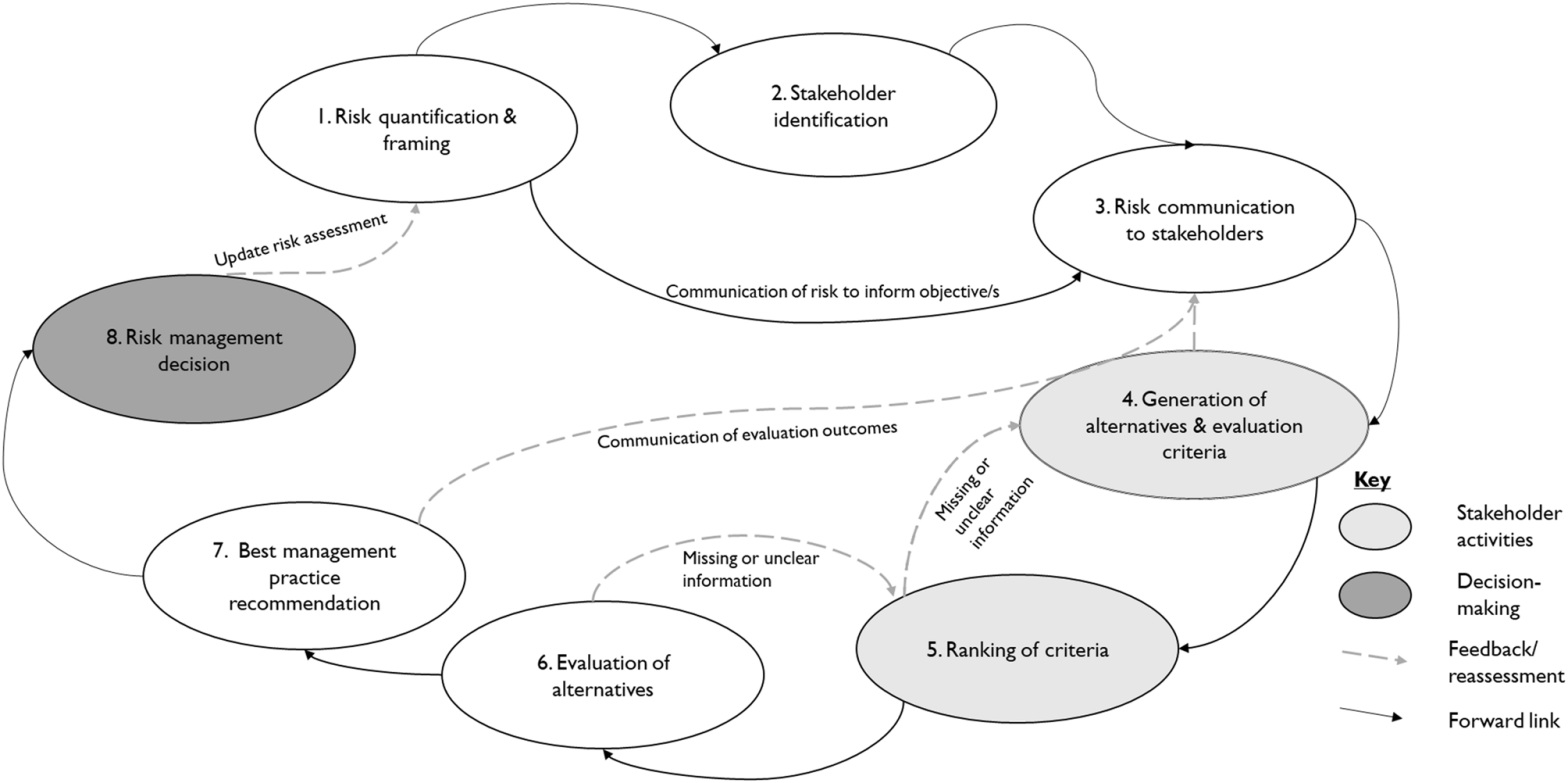
Conceptual framework for stakeholder engagement in the multi-criteria decision analysis process.

The methodology utilised in this study is illustrated in Fig. 2, which presents a conceptual framework for stakeholder engagement.

### MCDA and stakeholder involvement

The MCDA refers to the process of ranking and choosing between alternatives based on multiple criteria or objectives^18^. It is a method for systematically comparing the advantages and disadvantages of different alternatives in support of decision making^28^. Furthermore, some applications allocate budgets or other scarce resources among alternatives, to maximise efficiency^29^. Typically, MCDA process consists of the following phases^30^: (i) problem formulation including the identification of the objectives, criteria and measures for criteria and generation of alternatives; (ii) evaluation of the impacts of the alternatives and creation of a consequence table; (iii) integration of stakeholders' preferences and opinions on the significance of the objective and the weighting of the criteria; (iv) calculation of alternatives' total priorities using software such as Excel, for example; and (v) evaluation of the outcomes, including sensitivity analysis and recommendations.

The Simple Multi-Attribute Rating Technique for Enhanced Stakeholder Take-up (SMARTEST) is an MCDA methodology tailored by Bray^31^ to engage stakeholders in as many stages as possible during the decision-making process, without being onerous. The SMARTEST method was developed from the Simple Multi-Attribute Rating Technique Extended to Ranking (SMARTER) method developed by Edwards and Barron^32^. The SMARTER method employs a ranking approach to determine criteria weighting, which was considered less burdensome than the Swing method used in earlier versions of the method^32^. SMARTER uses the rank order centroid (ROC) method to convert ranks into weights, however Roberts and Goodwin^33^ argued that the rank order distribution (ROD) method is a better choice, even though it is complex to calculate. Furthermore, Roberts and Goodwin^33^ suggested the Rank-Sum (RS) method as a simpler approximation. In the SMARTEST, the ranks are therefore converted to weights using the RS method, which is simple and closely agrees with the ROC weighting method used in SMARTER^31^.

There were two stages to stakeholder consultation in this study (depicted as 4 & 5 in Fig. 2). Stage 1 was performed to solicit decision alternatives and evaluation criteria from stakeholders. In Stage 2 stakeholders were asked to refine the alternatives and criteria solicited from them in Stage 1, as well as to rank the criteria. The ranking of the criteria helps ensure that the decision-making process fairly reflects the preferences and values of all stakeholders involved^31^. The methodology delineated in Fig. 2 spanned a duration of eleven months. The data collection for stage 1 survey was conducted during November 2022-March 2023, while data collection for stage 2 survey was conducted during July–August 2023. The data from Stage 1 was analysed before Stage 2 commenced (April–May 2023) in order to share the findings with stakeholders during data collection for Stage 2.

A combination of stratified random sampling^34^ and purposive sampling^35^ was used in this study to ensure a representative sample. This approach aimed to capture diverse perspectives from stakeholders across different subbasins of the catchment and levels of involvement in river use and management. Stakeholders identified for participation in this study fall into the following groups as defined by the Department of Water Affairs of South Africa^36^:

- Affected parties: Those that are directly affected by the implementation of the strategy and its outcomes. In this study, these are communities within the uMsunduzi catchment, including professionals such as scientists, engineers, and athletes who use the river. Populace outside of this catchment, was excluded from this study in this category.
- Involved parties: Those involved in catchment management like local government, and those financially and legally involved. Representation of all involved municipalities, including the eThekwini (Durban) Metropolitan were considered in this study. Individuals in this category represented their respective organisations.
- Interested parties: Those who have an interest in broader developments (for example, environmentalists, other developers, and the interested academics. Individuals in this category represented their respective organisations.

The engagement of the identified stakeholders was performed partly with the guide of DWAF^36^, namely: (i) informing stakeholders, (ii) meeting with stakeholders, (iii) feedback to and from stakeholders, and (iv) monitoring and evaluation. The different stakeholder groups were informed, and ethical clearance was sought before commencement, as guided by DWAF^36^. The experimental protocol was approved by the local Tribal Councils, the South African Institute of Civil Engineering (SAICE), and the involved municipalities. The Gatekeeper Letter and the Letter of Consent were translated into isiZulu, which is the predominant local language, particularly in rural areas. Prior to data collection, informed consent was obtained from participants. This involved explaining the purpose of the survey, how the data will be used, and any potential risks or benefits associated with participation. The study was carried out in accordance with relevant guidelines and regulations.

### Development of decision alternatives and evaluation criteria (stage 1)

In Stage 1 of stakeholder engagement, the primary activities involved soliciting decision alternatives and evaluation criteria. These activities encompassed several key steps: first, requesting consent and obtaining the necessary Gatekeeper letters, with additional clarification provided as needed; followed by a concise presentation of the study to inform stakeholders about identified risks based on QMRA and QCRA findings^22,23^. Stakeholders were then provided with a user-friendly survey form, which they could complete at their own pace to ensure their comfort with the process.

This survey form was designed on the Microsoft Forms platform for ease of data collection and collation. The survey link was distributed to participants through materials shared during the meetings and, where applicable, via email. The survey form was designed to not automatically collect information such as names, addresses, and contact details through anonymisation to protect participants’ privacy. The raw data remains stored securely for five years and it is password protected to prevent unauthorised access and tampering. This data will be disposed of securely when it is no longer needed. This survey had nine questions, which were designed as a mix of short questions, Likert scale, multiple choice, and long answer questions. The specific questions are shown in Table 1. Where applicable and requested, the questions were translated to isiZulu during meetings, making the survey a structured interview instead.

**Table 1:** Questions in Stage 1 of stakeholder engagement survey.

Question	Options/sub-questions
1. Please select the stakeholder group to which you belong	Affected parties (resident/sport participant within uMsunduzi catchment) Involved parties (municipality) Interested parties (environmentalists, hydrologists, water utilities, NGOs, NPOs, etc.)
2. Contact details	
3. Please provide the name of the organisation that you represent or the name of the area you live in within the catchment	
4. Please select how you or anyone in your household use the river water. You may select multiple options	Bathing, Swimming, Cooking, Washing clothes, Discard waste, Canoeing, I do not use the river at all, Religious/cultural reasons, Other
5. Please indicate how important the following (indicators) factors are in deciding whether river water quality is good or bad. Colour, Taste, Smell, Presence of aquatic plants in the river, Presence of waste (faeces, plastics) around the riverbank or in the river	Likert options were: Not at all important, A little important, Neutral, Considerably important, Very important
6. How important is it to you that the water quality in the river improves?	Likert options were; Extremely important, Somewhat important, Neutral, Somewhat not important, Extremely not important
7. Please enlist possible strategies that you would like to see being adopted to improve water quality	
8. What is important to consider when selecting the best strategy? List any parameters that you think can be useful	
9. Please use this part to give any other thoughts you have at this stage based on the presented information and the questions above	

The results of the survey were analysed using Microsoft Excel and IBM SPSS version 29 for descriptive statistics, in preparation for Stage 2. The Relative Importance Index (RII) was used to assess the ranked degree of importance^37^. The RII of each factor *i* was calculated using (1 where *r*_*j*_ was jth respondents rating of the factor; A was the highest possible rating/ranking; and N was the number of respondents. RII (Eq. (1)) was used to analyse the responses to question 5 and 6 of Stage 1 survey.

1 $${RII}_{i}= \frac{{\sum }_{j}^{N}{r}_{j}}{A\times N}$$

### Ranking and weighing of evaluation criteria (stage 2)

The aim of Stage 2 survey was to provide feedback on Stage 1 survey to stakeholders and to solicit ranks for evaluation criteria from them. After Stage 1, responses to questions 7 and 8 in Table 1 were categorised, coded, themed, and collated for feedback to stakeholders during Stage 2. The stakeholders were given a chance to assess the themed alternatives and add what they feel was omitted or underrepresented or remove what they felt was irrelevant using question 4 in Stage 2 survey. The feedback to stakeholders was done over emails, telephone or in person meetings, based on the stakeholder preferences. The stakeholders were requested to rank the criteria in order of importance from highest to lowest. Table 2 shows the seven specific questions that were asked in Stage 2 of stakeholder engagement.

**Table 2:** Questions in Stage 2 of stakeholder engagement survey.

Question	Options/sub-questions
1. Please select the stakeholder group to which you belong	Affected parties (resident/sport participant within uMsunduzi catchment) Involved parties (municipality) Interested parties (environmentalists, hydrologists, water utilities, NGOs, NPOs, etc.)
2. Contact details	
3. Please provide the name of the organisation that you represent or the name of the area you live in within the catchment	
4. The following list represents the strategies for pollution alleviation that were listed by the stakeholders in stage 1. Please think about whether you would like to add or remove any of them and indicate that below	(i) Public education and outreach (Livestock grazing management and education on the impact of livestock on the river water and vice versa. Educate people on safe disposal of medication. Fertilizer and pesticide management) (ii) Fixing of sewer system (iii) Constructed wetlands iv) Runoff control (Through detention ponds, river buffer etc.) (v) Solid waste control (Through recycling, river cleanup etc.)
5. Please select the projects that would be acceptable to you if they were to be chosen as the best strategy. You may select more than one	(i) Public education and outreach (ii) Fixing of sewer system (iii) Constructed wetlands (iv) Runoff control (v) Solid waste control
6. When stakeholders were asked to select parameters for selecting the best strategy in stage 1, the following were listed. Please rank them from the most important to the least important	(a) Project funding/capital costs (costs of implementation, operation, and maintenance) (b) Socio-economic benefits (potential of job creation) (c) Feasibility (can it be done in this catchment?) (d) Community acceptance (acceptance by affected stakeholders) (e) Aesthetics (potential creation of scenic values) (f) Sustainability (pollutant load reduction/potential long-term effects)
7. If you wish to add more parameters for selecting the best strategy, please use the space below	

Questions 5 and 6 in Table 2 were analysed using the RII method as shown in Eq. (1 to get the ranks for each factor. Subsequently, to derive the weights from the ranks, the Rank Sum (RS) method was used in this study using Eq. (2). In this equation, *w*_i_ was the weight of the *i*th criteria of n, and r_*i*_ was its ranking as determined for question 6 in Table 2 using the RII value to rank the criteria^31^.

2 $${w}_{{\text{i}}}= \frac{2 (n + 1-{r}_{i})}{n(n+1)}$$

### Assessment of decision alternatives

The aggregate scores for decision alternatives were derived through Eq. (3)^31^.

3 $$v(a)=\sum {w}_{i} {v}_{i}(a)$$

where *v*(*a*) was the aggregate score of alternative *a*, *vi* (*a*) was the performance score of alternative *a* on criteria *i*, and *wi* was the weight of criterion *i* as derived from the RS method in Eq. (2).

The criteria used to assess the decision alternatives are shown in Table 3. The performance scores were compiled using a combination of literature and expert opinion. Literature was used to establish the cost ranges for the criterion “Project funding/capital cost”, and expert opinions were used to assign the performance scores for the criteria “Socio-economic benefits”, “Aesthetics”, “Sustainability”, “Feasibility”, for each decision alternative. The performance scores for the criterion “Community acceptance” were based on Stage 2 survey question 5 in Table 2. The criteria were classified into two categories: "benefit" and "cost". Under the “benefit” criterion, a score of 1 was assigned to low, 2 to medium, and 3 to high. Conversely, for the "cost" criteria, high was scored 1 and low was scored 3. Project funding is shown in South African Rands and converted to Euro and US Dollar in brackets based on exchange rates in August 2023 (1 US$ = R19, and 1€ = R20).

**Table 3:** Evaluation criteria used to assess the decision alternatives.

Score definition	Criteria definition	Performance score
Socio-economic benefits		
Low	Minimal increase in job creation	1
Medium	Moderate increase in job creation	2
High	Significant increase in job creation	3
Project funding/capital costs		
High	> R20M (1 070 583 USD) (980 088 Euro)	1
Medium	R10M-R20M	2
Low	< R10M (535 304 USD) (490 044 Euro)	3
Community acceptance		
Low	Low community support	1
Medium	Limited community support	2
High	Widespread community support	3
Aesthetics		
Low	No scenic value potential	1
Medium	Moderate scenic value potential	2
High	Significant scenic value potential	3
Sustainability		
Low	No noticeable reduction in pollution levels	1
Medium	Moderate reduction in pollution levels	2
High	Substantial and lasting reduction in pollution levels	3
Feasibility		
Low	Requires significant morphological changes	1
Medium	Achievable with some morphological advancements	2
High	Easily achievable in current catchment morphology	3

In the context of sensitivity analysis, the robustness of the results was assessed through examining how variations in the criteria ranks could affect the outcome in the decision-making model through scenario analysis. Stakeholders were not a part of this process. Nine random possible rank combinations (RC1-9) were considered as depicted in Supplementary Table S1. The resulting scores of the alternatives were compared with the scores from the original combinations.

### Ethics declarations

Informed consent was duly obtained from participants.

## Results

During the initial phase (Stage 1) of the study, 39 individuals took part, comprising three stakeholder categories. Specifically, there were 30 affected stakeholders, one representative from a municipality as an involved stakeholder, and eight interested stakeholders. Among the 30 affected stakeholders, nine were from rural areas, seven from the suburban areas, seven from townships, five from informal settlements next to an industrial park, one was a canoe club representative, and one was from an industrial park. Of the eight interested stakeholders, there were NGOs, university members, environmentalists, and civil engineers.

**Figure 3:**
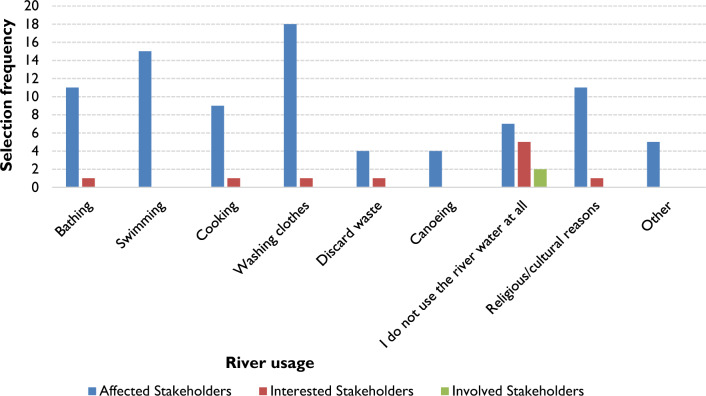
UMsunduzi River usage by stakeholders based on question 4 of survey Stage 1 (39 participants). The number of responses has been presented as the selection frequency on the y-axis expressed in terms of different stakeholders.

The data collected regarding the river's utilisation demonstrated that affected stakeholders depend on it for a range of activities, including cooking, washing clothes, bathing (the act of washing one’s body), swimming, and engaging in religious and cultural practices, as illustrated in Fig. 3. All participants responded to this question, with the affected stakeholders indicating a wide range of use, the involved stakeholder indicated no use of the river at all, and only two interested stakeholders indicated some use of the river. Moreover, under the "Other" category, affected stakeholders mentioned that their livestock also drink from the river.

**Figure 4:**
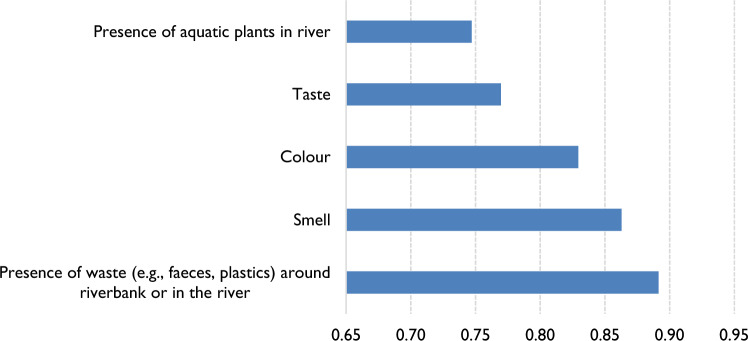
Relative importance index of physical characteristics of water to participants based on question 5 of survey Stage 1 (39 participants).

When stakeholders were requested to express the significance of certain indicators in determining the quality of river water, they chose the presence of waste in the water or on the riverbanks as the most important factor in deciding whether the quality of water is good or bad, as depicted in Fig. 4.

During the second phase (Stage 2) of the study, input was obtained from 21 participants, consisting of 17 affected stakeholders, one municipality representative as an involved stakeholder, and three interested stakeholders. Of the 17 affected stakeholders, five were from suburban areas, four from rural areas, five from townships, two from informal settlements, and one was a canoeist representing their canoeing club. Amongst the interested stakeholders, two were from NGOs and one was an environmental scientist representing their respective research and education institution. While the total number of participants decreased in the second stage, the different stakeholder groups were still well-presented.

In Stage 2, stakeholders were presented with a list of alternatives that were identified during Stage 1. When stakeholders were asked to add or remove any of the alternatives (Question 4 of Stage 2 survey), for inclusion, they listed: “*Landowner stewardship; Erosion control measures; Maintenance and management of roads; Livestock exclusion from streambeds; Maintenance of riparian zones to serve as buffers; and Compliance monitoring checks”.* No one wanted to remove anything from the list.

Stakeholders were asked in Stage 1 to propose criteria that can be used to evaluate the alternatives. The criteria were grouped as economic, environmental, and social. Overall, six sub-criteria were presented for ranking. Table 4 shows the criteria ranks given by stakeholders and the subsequent weightings deduced using the Rank Sum method (2).

**Table 4:** Ranking of evaluation criteria by stakeholders in Stage 2 (21 participants) and the corresponding weights derived through Rank Sum method.

Criterion	Ranking	Weighting
Project funding/capital costs (costs of implementation, operation, and maintenance)	1	0.2857
Feasibility (can it be done in this catchment?)	2	0.2381
Socio-economic benefits (potential of job creation)	3	0.1905
Community acceptance (acceptance by affected stakeholders)	4	0.1429
Sustainability (pollutant load reduction/potential long-term effects)	5	0.0952
Aesthetics (potential creation of scenic values)	6	0.0476

To assess the performance of each decision alternative on each evaluation criterion (Table 3), a performance matrix was formulated, as detailed in Table 5. Public education and outreach as well as runoff control scored lower than the other alternatives on the socio-economic benefit criterion. Fixing the sewers was the only alternative with a high capital cost, while the other alternatives were deemed inexpensive and scored low on this criterion. Most alternatives scored high on community acceptance and environmental criteria, except for constructed wetlands and runoff control, while constructed wetlands was the only alternative scoring high on aesthetics.

**Table 5:** Performance matrix for decision alternatives for the defined criteria.

Decision alternative	Economic criteria		Social criteria		Environmental criteria	
	Socio-economic benefits	Project funding/capital costs	Community acceptance	Aesthetics	Sustainability	Feasibility
Public Education and Outreach	Medium (2)	Low (3)	High (3)	Medium (2)	High (3)	High (3)
Fixing of sewer systems	High (3)	High (1)	High (3)	Medium (2)	High (3)	High (3)
Solid waste control	High (3)	Low (3)	High (3)	Medium (2)	High (3)	High (3)
Constructed wetlands	High (3)	Low (3)	Low (1)	High (3)	Medium (2)	Medium (2)
Runoff control	Medium (2)	Low (3)	Low (1)	Medium (2)	Medium (2)	Medium (2)

Table 6 illustrates the aggregated scores (Eq. (3)) that account for the weight of the criteria for the decision alternatives, revealing that solid waste control emerges as the top-performing BMP, closely followed by public education and outreach, with sewer system repairs as well as constructed wetlands coming in third. These aggregated scores, based on the performance score of each alternative and the weight of each criterion, indicate the importance of non-structural BMPs that influence behavioural change within this catchment. In the sensitivity analysis of the MCDA model, the impact of varying criteria ranks on the final scores of the decision alternatives was systematically assessed (Table 6). Public education and outreach as well as solid waste control are in the top three alternatives for all sensitivity scenarios, reinforcing the results of the analysis. Solid waste control was the top alternative and runoff control was the bottom alternative in all scenarios, that is, irrespective of the criteria ranks.

**Table 6:** Aggregated scores of decision alternatives without criteria weights, alongside weights assigned based on stakeholder ranking, and sensitivity analysis of criteria ranking.

Decision alternatives	Without weights	With weights	Sensitivity analysis of criteria ranking (weights)								
			RC 1	RC 2	RC 3	RC 4	RC 5	RC 6	RC 7	RC 8	RC 9
Public education and outreach	2.7	2.8	2.8	2.7	2.8	2.8	2.5	2.7	2.7	2.8	2.6
Fixing of sewer systems	2.5	2.4	2.5	2.6	2.7	2.8	2.6	2.3	2.3	2.2	2.2
Solid waste control	2.8	3.0	3.0	3.0	3.0	3.0	2.7	2.9	2.9	2.8	2.8
Constructed wetlands	2.3	2.4	2.3	2.4	2.1	2.2	2.4	2.4	2.6	2.4	2.6
Runoff control	2.0	2.1	2.1	2.0	1.9	2.0	1.9	2.1	2.2	2.1	2.1

## Discussion

In the context of the uMsunduzi catchment in South Africa, this research sought to offer watershed managers support for the selection of BMPs aimed at mitigating pollution and associated risks to river users. This was achieved by applying the comprehensive framework (Fig. 2) that incorporates the valuable perspectives of catchment stakeholders. In this study a framework for stakeholder engagement in pollution alleviation and risk management was proposed and exemplified for uMsunduzi River in South Africa. This focus on stakeholder involvement in decision-making, was advocated by Sharpe et al.^38^ to underscore the significance of local community participation in preventing project immobilisation and improving decision-making. This approach further aligns with the Department of Water Affairs of South Africa's^36^ categorisation of water resource management stakeholders into affected, involved, and interested parties, prioritising those directly affected by development projects, local government, and stakeholders with broader interests. Furthermore, Sharpe et al.^38^ proposed comprehensive criteria for stakeholder selection, considering factors such as level of interest, influence, impact, probability, proximity, economic interest, rights, fairness, and underrepresented populations. In the current study, this DWAF approach provides a contextually appropriate and detailed stakeholder categorisation. However, there are limitations related to stakeholder participation, evident from the reduced number of participants in Stage 2, which may reflect their areas of interest and available time, emphasising the need to balance stakeholder expectations with project timelines. Nevertheless, the achieved sample size, coupled with the sampling strategies implemented, facilitated a multifaceted comprehension of stakeholder perspectives. This exemplifies the breadth and depth of insights necessary to achieve the study's objectives.

Care was taken in the study's approach towards stakeholder involvement by ensuring that participants could engage comfortably by offering survey instructions in English and facilitating discussions with stakeholders in rural areas, townships, and informal settlements in isiZulu, fostering a deeper understanding of scientific evidence and instructions. This approach aligns with local cultural context and enhances inclusivity as emphasized by Behr^39^. If this methodology were applied on a larger scale, translating all materials into the relevant language would be advisable to further enhance understanding and response rates, ultimately promoting more effective and meaningful stakeholder participation. Although this study maintained isolation between different stakeholder groups, future research could benefit from integrating workshops guided by a moderator in the consultation process. Workshops have the potential to expose participants to new information and perspectives from other stakeholders, broadening their views and positively influencing their preferences, in line with findings by Marttunen et al*.*^40^. Additionally, setting up a structured feedback mechanism from stakeholders to assess their perceptions of the process, as suggested by Lück and Nyga^16^, can further enhance the engagement approach.

The Stage 1 survey findings shed light on the factors that respondents consider most critical in assessing water quality. Notably, solid waste and faecal matter emerged as the top concerns, with taste ranking as the least important in their collective assessment. This aligns with the research conducted by Okumah et al.^41^ in Ghana, which underscores the significance of clean and hygienic surroundings for water resources. In such environments, free from solid and liquid waste, water is more likely to be perceived as safe for consumption and recreational activities. However, a contrasting perspective was revealed by Rangecroft^42^ in their study of water quality perceptions in the Santa basin, Peru. Their research highlighted the pivotal role of organoleptic properties, such as taste, smell, and visual aspects, in shaping local perceptions of water quality, along with traditional ecological knowledge and water usability. This striking difference underscores the notion that local perceptions of water quality are intricately linked to the specific uses and cultural contexts of the water.

In the Stage 1 survey, Likert scales were used to assess the factors influencing river water quality (Question 5) and the importance of improving water quality (Question 6). The Likert scales differed due to oversight during the survey creation and during the piloting of the survey. Despite diverse stakeholder perspectives on river utilisation and the identified inconsistency with the questions, all participants unanimously agreed on the river's inadequate condition, highlighting a consensus on the urgency of improving water quality. The possible alternatives brought up by stakeholders were public education and outreach, fixing of sewer system, constructed wetlands, runoff control, and solid waste control. The identification of alternatives for the decision problem should be based on case-specific information^43^. Authors judge that the stakeholder responses in the uMsunduzi case all provide potentially sustainable solutions that can satisfy the problem objectives. Even though there may be additional alternatives that would meet the objectives, the authors concluded that the derived alternatives presented a wide enough range of possible solutions. The evaluation criteria selected by stakeholders could be grouped as project funding / capital costs (costs of implementation, operation, and maintenance), socio-economic benefits (potential of job creation), feasibility (can it be done in this catchment?), socio-economic benefits (potential of job creation), sustainability (pollutant load reduction/potential long-term effects), and aesthetics (potential creation of scenic values).

Solid waste management emerged as the preferred BMP, aligning with the prominent water quality indicator, presence of waste (faeces, plastics) around the riverbank or in the river, identified by stakeholders in Stage 1. Effective solid waste management, sometimes challenging^44,45^, can be an important part in mitigating water pollution^45–47^. Challenges include increasing waste volumes, financial constraints, limitations within existing containment systems^44^ as well as limited public awareness about recycling, a shortage of engineering expertise in waste management, substandard service delivery, and a lack of effective educational campaigns^45^. In essence, while addressing solid waste is important to promote environmentally friendly practices, it is essential to recognise that dealing with chemical and microbial pollution requires additional efforts beyond solid waste management alone.

Interestingly, the second management plan was public education and awareness, highlighted as vital for addressing water pollution^46^. Osawe et al.^48^ proposed community-based initiatives for behavioural change to address declining water quality. This aligns with the study by Brehm and Eisenhauer^49^, emphasising the positive impact of public education in pollution mitigation, underscoring the growing importance of community engagement and collaboration in addressing water pollution. It is imperative for public education to encompass subjects such as livestock management, given its established role in faecal pollution and the resulting microbial health risks for humans^22^. Moreover, the proper disposal of medical waste requires control and public education can play a significant role, possibly with involvement from the pharmaceutical industry.

Another prominent management plan involves repairing and maintaining the sewer systems. Research has emphasised insufficient wastewater treatment plant performance in South Africa, underscoring negative impact on the environmental and public health consequences^50,51^. In Ngubane et al.^52^ human faecal sources were found to be more prominent in urban areas owing to the major contributions from wastewater infrastructure. A report by South African Institute of Civil Engineering^53^ further emphasised the pressing need for sewer system improvements, as 34% of sanitation systems face high or critical risk of failure. South Africa's water infrastructure challenges are evident, with just 40% of wastewater effluent meeting microbial water quality standards and 23% of wastewater effluent meeting chemical water quality standards^53^. Specifically, the uMsunduzi sewer pipeline, which spans over 1450 kms, has aged considerably, with approximately 60% of it being between 30 to 50 years old^21^.

Constructed wetlands mimic natural ecosystems in purifying water, preserving water quality, and providing habitats for wildlife and recreation^46^. Research has shown that constructed wetlands efficiently remove a wide range of pollutants, including nutrients, heavy metals, organic compounds, and PPCPs^54^. These pollutants have been detected in the uMsunduzi River with their human health risk quantified by Ngubane et al*.*^23^. The last management plan involves runoff control measures, including detention ponds, designed to reduce stormwater peak flow and capture sediment and nutrients^5,55^. Key processes for reducing pollutants are sedimentation and biological processes^5^, and these ponds help manage stormwater surges by delaying runoff to nearby rivers^55^. The effectiveness depends on factors like size, retention time, and local rainfall intensity^55^. Detention ponds have been reported to reduce total nitrogen, nitrate, ammonium, total phosphorus, orthophosphate, and faecal coliform bacterial counts^5,55^.

The order of the decision alternatives with and without accounting for stakeholder ranking of the evaluation criteria is very similar (Table 6). Moreover, the sensitivity analysis demonstrated that while the top (solid waste management) and the bottom (runoff control) alternatives remained the same, the order of the other alternatives may change depending on stakeholder ranking of the criteria. As suggested by Giupponi and Sgobbi^56^, when variations in factor weights during sensitivity analysis do not result in substantial alterations in the model's outcomes, it can be inferred that the model yields more objective and consistent results. In this study the top alternative remains the same, regardless of sensitivity scenarios. Despite the acknowledged limitations of the case study, the model offers a comprehensive perspective on the impacts being examined.

The choice of solid waste management as the stakeholders’ primary decision alternative aligns with its immediate practical importance, while the emphasis on education (stakeholders’ secondary choice to improve the uMsunduzi water quality) acknowledges the need for a sustainable, long-term solution. These results should guide the development of comprehensive BMPs that combine practical infrastructure improvements with educational initiatives^57^. Additionally, broadening the stakeholder base and refining the methodology through using workshops for stakeholder engagement could further enhance decision-making in this context. Future use of this framework within the study area should consider if criteria and alternatives proposed here are complete, or whether additional criteria/alternatives need to be developed.

The choice of stakeholders, their representation, and the level of their engagement can influence the weighting of criteria, the evaluation of alternatives, and the final decision outcomes^58^, or even the choices of criteria and alternatives. To overcome parts of subjectivities and provide less biased information, one option is to integrate cost–benefit analysis in future studies to diversify and broaden the available decision support^59^. This addition, as presented in, for example, Bergion^60^, could provide more comprehensive economic criteria for MCDA and facilitate comparisons between different mitigation measures. Specifically, the use of social cost–benefit analysis was suggested to assess the societal benefits of individual projects and identify the most economically viable pollution alleviation strategies^60^. The social cost–benefit analysis additionally considers the non-financial effects^60^.

The current study introduces a novel risk management framework, depicted in Fig. 2, which fills a crucial gap in literature^61^ by integrating risk assessment within the MCDA framework for watershed-scale pollution. This innovative approach lays the groundwork for a more holistic and robust strategy in pollution risk management. With the focus on developing countries with diverse land uses within catchments, the framework underwent practical testing in the uMsunduzi catchment, South Africa, with potential global applicability. To ensure adaptability and transferability, key aspects such as assessing data availability and collaborating with local stakeholders are paramount. Furthermore, the framework's flexibility allows for accommodation of environmental variations and regulatory frameworks.

## Conclusions

In the context of the uMsunduzi River catchment in South Africa, this study aimed to provide a framework for watershed managers to select effective BMPs for pollution reduction, addressing the associated risks to human health, while incorporating the perspectives of key stakeholders. Multi-criteria decision analysis using the SMARTEST method proved to be a valuable tool for enhancing stakeholder engagement in collaborative risk management. Furthermore, the study's outcomes are based on the perspectives of stakeholders at the time of data collection and may evolve over time. These limitations highlight the need for tailored approaches when applying the study's recommendations in other regions or considering long-term sustainability.

The MCDA results revealed that solid waste control emerged as the top-ranked BMP, closely followed by public education and outreach. This study provides a valuable novel approach for informed decision-making in water quality management incorporating local stakeholder perspectives, with the potential for broader application in similar contexts. The framework offers a robust foundation for developing strategies to enhance river water quality and reduce pollution, emphasising the importance of stakeholder involvement, effective BMP selection, and health risk management in environmental sustainability efforts.

### Supplementary Information

Supplementary Table S1.

## Data Availability

The data that support the findings of this study are available from the Durban University of Technology but restrictions apply to the availability of these data, which were used under license for the current study, and so are not publicly available. Data are however available from the corresponding authors upon reasonable request and with permission of the Durban University of Technology.

## References

[1] Anawar HM, Chowdhury R (2020). Remediation of polluted riverwater by biological, chemical, ecological and engineering processes. Sustain..

[2] Cullis JDS (2019). Urbanisation, climate change and its impact on water quality and economic risks in a water scarce and rapidly urbanising catchment: Case study of the Berg River Catchment. H2Open J..

[3] da Silva Bonifácio A (2021). Human health risk assessment of metals and anions in surface water from a mineral coal region in Brazil. Environ. Monit. Assess..

[4] Shortle JS, Mihelcic JR, Zhang Q, Arabi M (2020). Nutrient control in water bodies: A systems approach. J. Environ. Qual..

[5] Rohrer A (2014). Cape Town’s Ponds: Urban Water Manegement.

[6] Aladesote OJ (2022). Cost-benefit analysis of green infrastructure for sustainable stormwater management of the built environment. Int. J. Res. Publ..

[7] WHO (2013). Animal waste, water quality and human health. Water Intell. Online.

[8] Shao H (2017). An open source GIS-based decision support system for watershed evaluation of best management practices. J. Am. Water Resour. Assoc..

[9] Lam QD, Schmalz B, Fohrer N (2011). The impact of agricultural Best Management Practices on water quality in a North German lowland catchment. Environ. Monit. Assess..

[10] Shastri H, Salvi K, Kulkarni S, Misra S (2021). Water and Energy Management in India. Water and Energy Management in India.

[11] Wu L (2022). Efficiency assessment of best management practices in sediment reduction by investigating cost-effective tradeoffs. Agric. Water Manag..

[12] Jing L, Chen B, Zhang B, Li P, Zheng J (2013). Monte Carlo simulation–aided analytic hierarchy process approach: Case study of assessing preferred non-point-source pollution control best management practices. J. Environ. Eng..

[13] Marttunen M, Mustajoki J, Lehtoranta V, Saariskoski H (2022). Complementary use of the ecosystem service concept and multi-criteria decision analysis in water management. Environ. Manag..

[14] Schreiner B (2013). Viewpoint—Why has the South African national water act been so difficult to implement?. Water Altern..

[15] Adom RK, Simatele MD (2022). The role of stakeholder engagement in sustainable water resource management in South Africa. Nat. Resour. Forum.

[16] Lück A, Nyga I (2018). Experiences of stakeholder participation in multi-criteria decision analysis (MCDA) processes for water infrastructure processes for water infrastructure. Urban Water J..

[17] Ndeketeya A, Dundu M (2022). Alternative water sources as a pragmatic approach to improving water security. Resour. Conserv. Recycl. Adv..

[18] Razmak J, Aouni B (2014). Decision support system and multi-criteria decision aid: A state of the art and perspectives. J. Multi-Criteria Decis. Anal..

[19] Simpungwe E (2006). Water, Stakeholders, and Common Ground: Challenges for Multi-Stakeholder Platforms in Water Resource Management, South Africa.

[20] Adeyinka, G. C., Moodley, B., Birungi, G. & Ndungu, P. Evaluation of organochlorinated pesticide (OCP) residues in soil, sediment and water from the Msunduzi River in South Africa. *Environ. Earth Sci.* **78**, 0 (2019).

[21] Msunduzi Municipality. *Draft integrated development plan 2022–2027*. https://www.mbombela.gov.za/approved draft idp for 2022–2027 financial years.pdf (2022).

[22] Ngubane Z (2022). Water quality modelling and quantitative microbial risk assessment for uMsunduzi River in South Africa. J. Water Health.

[23] Ngubane Z, Dzwairo B, Moodley B, Stenström TA, Sokolova E (2023). Quantitative assessment of human health risks from chemical pollution in the uMsunduzi River, South Africa. Environ. Sci. Pollut. Res..

[24] STATSSA. *General household survey 2021*. *Department of Statistics South Africa.* Vol. 21. http://www.ncbi.nlm.nih.gov/pubmed/11469378 (2022).

[25] Arnold *et al.* Soil & Water Assessment Tool. (2012).

[26] Haas CN, Rose JB, Gerba CP (2014). Quantitative Microbial Risk Assessment.

[27] Umgeni Water. *Infrastructure master plan 2022–2053*. Vol. 2. https://www.umgeni.co.za/wp-content/uploads/2022/07/UWIMP_2022_Vol2.pdf (2022).

[28] Esmail BA, Geneletti D (2018). Mutli-criteria decision analysis for nature conservation: A review of 20 years of applications. Methods Ecol. Evol..

[29] Prato T, Herath G (2007). Multiple-criteria decision analysis for integrated catchment management. Ecol. Econ..

[30] Saarikoski H (2016). Multi-criteria decision analysis and cost-benefit analysis: Comparing alternative frameworks for integrated valuation of ecosystem services. Ecosyst. Serv..

[31] Bray R (2015). Developing a participative multi criteria decision making technique: A case study. Int. J. Manag. Decis. Mak..

[32] Edwards W, Barron FH (1994). Smarts and smarter: Improved simple methods for multiattribute utility measurement. Organ. Behav. Hum. Decis. Process..

[33] Roberts RON, Goodwin P (2003). Weight approximations in multi-attribute decision models. J. Multi-Criteria Decis. Anal..

[34] Singh AS, Masuku MB (2011). Sampling techniques & determination of sample size in applied statistics research. Inwood Mag..

[35] Rai N, Thapa B (2019). A study on purposive sampling method in research. KathmanduKathmandu Sch. Law.

[36] DWAF. *Water quality management series: A guide to stakeholder identification and involvement*. https://www.gov.za/documents/other/managing-water-quality-effects-settlements-national-strategy-01-apr-1999.

[37] Tholibon DA (2021). Relative Importance Index (RII) in ranking the factors of employer satisfaction towards industrial training students. Int. J. Asian Educ..

[38] Sharpe LM, Harwell MC, Jackson CA (2021). Integrated stakeholder prioritization criteria for environmental management. J. Environ. Manag..

[39] Behr D (2018). Translating questionnaires for cross-national surveys: A description of a genre and its particularities based on the ISO 17100 categorization of translator competences. Transl. Interpret..

[40] Marttunen M, Mustajoki J, Dufva M (2013). How to design and realize participation of stakeholders in MCDA processes? A framework for selecting an appropriate approach. Eur. J. Decis. Process.

[41] Okumah M, Yeboah AS, Bonyah SK (2020). What matters most? Stakeholders’ perceptions of river water quality. Land Use Policy.

[42] Rangecroft S (2023). Unravelling and understanding local perceptions of water quality in the Santa basin, Peru. J. Hydrol..

[43] Freitas AHA, Magrini A (2013). Multi-criteria decision-making to support sustainable water management in a mining complex in Brazil. J. Clean. Prod..

[44] Idowu IA (2019). An analyses of the status of landfill classification systems in developing countries: Sub Saharan Africa landfill experiences. Waste Manag..

[45] Ayeleru OO, Okonta FN, Ntuli F (2021). Cost benefit analysis of a municipal solid waste recycling facility in Soweto, South Africa. Waste Manag..

[46] Sahoo SK, Goswami SS (2024). Theoretical framework for assessing the economic and environmental impact of water pollution: A detailed study on sustainable development of India. J. Future Sustain..

[47] Bangani L, Kabiti HM, Amoo O, Nakin MDV, Magayiyana Z (2023). Impacts of illegal solid waste dumping on the water quality of the Mthatha River. Water Pract. Technol..

[48] Osawe OW, Grilli G, Curtis J (2023). Community-funded behavioural change initiatives: Water quality in Ireland. Environ. Dev..

[49] Brehm JM, Eisenhauer BW (2021). Impacts of targeted education programs on the adoption of residential best management practices (BMP) to combat non-point source pollution. Appl. Environ. Educ. Commun..

[50] du Plessis A, du Plessis A (2023). South Africa’s water predicament: Freshwater’s unceasing decline. South Africa’s water predicament: Freshwater’s unceasing decline.

[51] du Plessis A, du Plessis A (2019). Water as an inescapable risk. Springer Water.

[52] Ngubane Z, Bergion V, Sokolova E (2022). Evaluating the health risks associated with drinking uMsunduzi River water, South Africa. IWA-WHO Int. Conf. Water Saf..

[53] SAICE. *SAICE 2022 Infrastructure report card for South Africa:* http://saice.org.za/downloads/saice-reportcard.pdf (2022).

[54] Salah M (2023). Insight into pharmaceutical and personal care products removal using constructed wetlands: A comprehensive review. Sci. Total Environ..

[55] Kaini P, Artita K, Nicklow JW (2012). Optimizing structural best management practices using SWAT and genetic algorithm to improve water quality goals. Water Resour. Manag..

[56] Giupponi C, Sgobbi A (2013). Decision support systems for water resources management in developing countries: Learning from experiences in Africa. Water (Switzerland).

[57] Bergion V, Lindhe A, Sokolova E, Rosén L (2020). Accounting for unexpected risk events in drinking water systems. Expo. Heal..

[58] Intelisano J, Lima ML, Veras N, Corleto B, Massone HE (2022). A multi-voiced model for decision-making in water resource management. A case study in the urban area of Mar del Plata city, Argentina. Urban Water J..

[59] Sjöstrand K, Lindhe A, Söderqvist T, Rosén L (2018). Sustainability assessments of regional water supply interventions—Combining cost-benefit and multi-criteria decision analyses. J. Environ. Manag..

[60] Bergion V, Lindhe A, Sokolova E, Rosén L (2018). Risk-based cost-benefit analysis for evaluating microbial risk mitigation in a drinking water system. Water Res..

[61] Akdogan Z, Guven B (2023). Multi-criteria decision analysis in assessing watershed scale pollution risk: A review of combined approaches and applications. Environ. Rev..

