# Supplementary material for: Multi-criteria decision analysis framework for engaging stakeholders in river pollution risk management
Source: Sci Rep. 2024 Mar 26;14:7125. doi: 10.1038/s41598-024-57739-y (PMC10966078; doi:10.1038/s41598-024-57739-y)
Supplement: Supplementary file 1 — Supplementary Table S1. [file 41598_2024_57739_MOESM1_ESM.docx]

# Multi-criteria decision analysis framework for engaging stakeholders in river pollution risk management

# Zesizwe Ngubane1, Viktor Bergion2,*, Bloodless Dzwairo1,3, Thor Axel Stenström3 and Ekaterina Sokolova4

1Durban University of Technology, Department of Civil Engineering, Pietermaritzburg, 3201, South Africa

2Chalmers University of Technology, Department of Architecture and Civil Engineering, Gothenburg, 41291, Sweden

3Durban University of Technology, Institute for Water and Wastewater Technology, Durban, 4000, South Africa

4Uppsala University, Department of Earth Sciences, Uppsala, 75105, Sweden

*[viktor.bergion@chalmers.se](mailto:viktor.bergion@chalmers.se)

**Supplementary Table S 1.** Rank combinations (RC) used for sensitivity analysis.

| **Criteria** | **Original** | **RC 1** | **RC 2** | **RC 3** | **RC 4** | **RC 5** | **RC 6** | **RC 7** | **RC 8** | **RC 9** |
| --- | --- | --- | --- | --- | --- | --- | --- | --- | --- | --- |
| Project funding/capital costs | 1 | 2 | 3 | 4 | 5 | 6 | 1 | 1 | 1 | 1 |
| Feasibility | 2 | 1 | 2 | 2 | 2 | 2 | 2 | 2 | 2 | 6 |
| Socio-economic benefits | 3 | 3 | 1 | 3 | 3 | 3 | 3 | 3 | 6 | 3 |
| Community acceptance | 4 | 4 | 4 | 1 | 4 | 4 | 4 | 6 | 4 | 4 |
| Sustainability | 5 | 5 | 5 | 5 | 1 | 5 | 6 | 5 | 5 | 5 |
| Aesthetics | 6 | 6 | 6 | 6 | 6 | 1 | 5 | 4 | 3 | 2 |
